# Inhibition of ERβ Induces Resistance to Cisplatin by Enhancing Rad51–Mediated DNA Repair in Human Medulloblastoma Cell Lines

**DOI:** 10.1371/journal.pone.0033867

**Published:** 2012-03-16

**Authors:** Anna Wilk, Agnieszka Waligorska, Piotr Waligorski, Augusto Ochoa, Krzysztof Reiss

**Affiliations:** 1 Neurological Cancer Research, Department of Medicine, LSU Health Sciences Center, New Orleans, Louisiana, United States of America; 2 Stanley S. Scott Cancer Center, Department of Medicine, LSU Health Sciences Center, New Orleans, Louisiana, United States of America; The Chinese University of Hong Kong, Hong Kong

## Abstract

Cisplatin is one of the most widely used and effective anticancer drugs against solid tumors including cerebellar tumor of the childhood, Medulloblastoma. However, cancer cells often develop resistance to cisplatin, which limits therapeutic effectiveness of this otherwise effective genotoxic drug. In this study, we demonstrate that human medulloblastoma cell lines develop acute resistance to cisplatin in the presence of estrogen receptor (ER) antagonist, ICI182,780. This unexpected finding involves a switch from the G2/M to G1 checkpoint accompanied by decrease in ATM/Chk2 and increase in ATR/Chk1 phosphorylation. We have previously reported that ERβ, which is highly expressed in medulloblastomas, translocates insulin receptor substrate 1 (IRS-1) to the nucleus, and that nuclear IRS-1 binds to Rad51 and attenuates homologous recombination directed DNA repair (HRR). Here, we demonstrate that in the presence of ICI182,780, cisplatin-treated medulloblastoma cells show recruitment of Rad51 to the sites of damaged DNA and increase in HRR activity. This enhanced DNA repair during the S phase preserved also clonogenic potential of medulloblastoma cells treated with cisplatin. In conclusion, inhibition of ERβ considered as a supplemental anticancer therapy, has been found to interfere with cisplatin–induced cytotoxicity in human medulloblastoma cell lines.

## Introduction

Medulloblastomas are the most common and aggressive intracranial tumors in children [1], [2], [3]. They originate from poorly differentiated neurons of the external granule layer of the cerebellum and have intrinsic propensity of spreading in CNS via subarachnoid spaces [4], [5], [6]. The most common clinical modalities against medulloblastoma include combination of radiation therapy (ranging from 20 to 55 Gy) and chemotherapy, which depending on the severity of the disease may consist of cisplatin or carboplatin supplemented by lomustine, and/or vincristine [3]. Despite of relatively good outcome of these therapies and 3-year progression-free survival rate for those adjuvant chemotherapies reaching almost 80% [3], recurrent medulloblastomas still represent a serious medical challenge. Recent detection of estrogen receptor β (ERβ) during development of the cerebellum [7], and its abundant expression in medulloblastoma clinical samples and in medulloblastoma cell lines [8], [9] implicates this nuclear receptor in normal development, however it also suggests its role in malignant transformation and possibly tumor progression [10], [11], [12], [13], [14]. Indeed, it has been recently reported that activation of ERβ in human medulloblastoma cell lines increased cell growth and cell migration [8], and ER antagonist, ICI182,780, inhibited medulloblastoma tumor growth in subcutaneous D283Med nude mouse model [8]. In addition, we have recently demonstrated that high-levels of ERβ in medulloblastoma are associated with nuclear translocation of insulin receptor substrate 1 (IRS-1), and the involvement of nuclear IRS-1 (nIRS-1) in the inhibition of homologous recombination directed DNA repair (HRR) of double strand breaks (DSBs). This interference with the DNA repair process involves a direct interaction between nIRS-1 and the major enzymatic component of HRR, Rad51 [9]. In this experimental model, inhibition of ERs by ICI182,780 repressed IRS-1 nuclear translocation and improved contribution of HRR in the process of DNA repair of DSBs [9]. Therefore, we conclude that ERβ, in addition to its supporting role in medulloblastoma cell growth and cell motility, interferes also with DNA repair of DSBs. This information could be relevant in view of recently proposed anti-ERβ strategy as a supplemental treatment against Medulloblastomas [8], [9]. Our present study demonstrates, however, that inhibition of ERβ by ICI182,780 may be associated with undesirable side effect. It triggers resistance of human medulloblastoma cell lines to cisplatin. This unexpected effect involves a switch from the G2/M to G1 phase checkpoint accompanied by the transition from ATM/Chk2 to ATR/Chk1 pathway, and better cell survival. In addition, we have detected elevated formation of Rad51 nuclear foci and significantly higher levels of HRR in the population of cells, which replicate DNA during the combined treatment of cells with cisplatin and ICI182,780. This new finding indicates that ICI182,780, by improving HRR, allows more effective repair of cisplatin-inflicted DNA damage during the S phase, which may explain decrease in G2/M arrest, improved cell survival, and partial preservation of the clonogenic growth of Daoy cells after removal of the genotoxic agent.

## Results

### Inhibition of ERβ correlates with better cell survival in the presence of cisplatin

**Figure 1:**
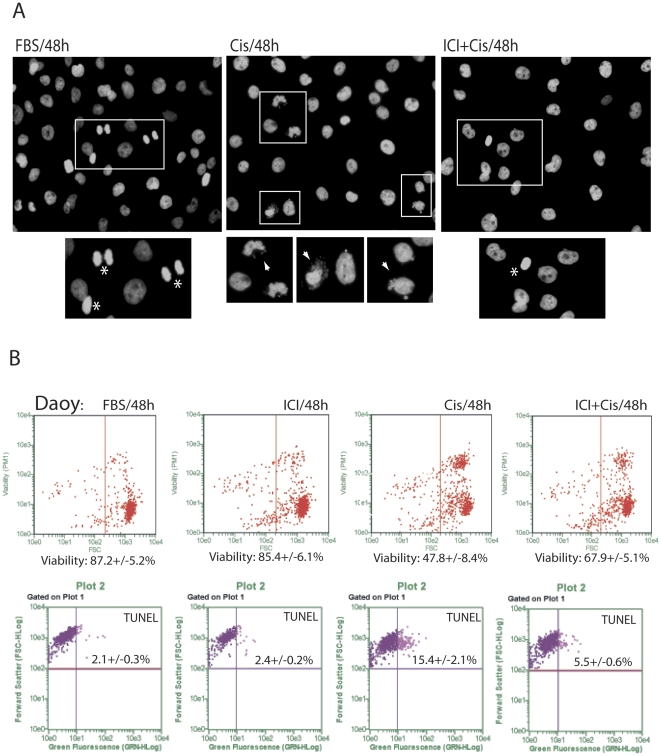
Inhibition of ERβ improves cell survival in the presence of cisplatin. **Panel A:** Fluorescent images showing nuclear morphology following labeling of DNA by fluorescent dye 4′,6-diamidino-2-phenylindole (DAPI). Exponentially growing monolayer cultures of Daoy cells (10%FBS) were treated with cisplatin (1 µg/ml) or with cisplatin + ICI182,780 (10 µM) for 48 hours. The images were taken with Nikon Eclipse 400 upright fluorescent microscope equipped with the motorized Z-axis, EXI-Aqua camera and deconvolution software (SlideBook5). Rectangles indicate magnified area containing cells in mitosis (asterix); and cells with damaged nuclei (arrowhead). Note that abundant presence of micronuclei (arrow) and nuclear fragmentation in cisplatin, and much less of the nuclear damage in cells treated by cisplatin+ICI182,780. Original magnification ×20. **Panel B:** Daoy cell viability evaluated by ViaCount and TUNEL assays. Both assays were adopted for the use with the GUAVA easyCyte 8HT flowcytometer (Millipore). The Guava/Express Plus and Guava/ViaCount software were used for data analysis and quantification according to the manufacturer recommendations (Millipore).

**Figure 2:**
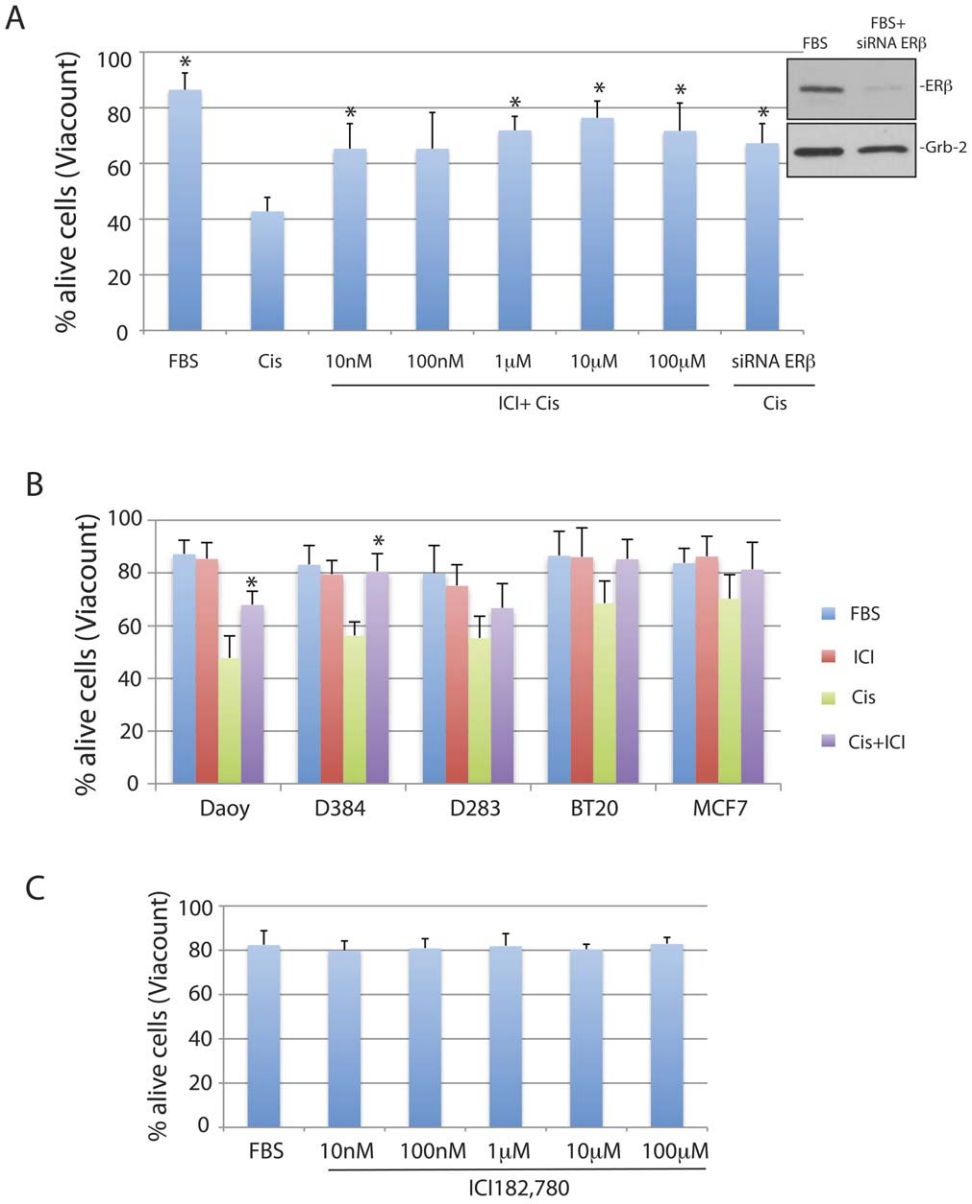
ICI182,780 dose response and tumor cell survival. **Panel A:** Evaluation of cell viability (ViaCount) of exponentially growing Daoy cells (FBS) treated with cisplatain (Cis; 1 µg/ml for 48 hrs) in the presence or absence of ERβ antagonist, ICI182,780 at indicated concentrations. In one instance the cells were preincubated for 48 hrs with siRNA against ERβ mRNA (siRNA ERβ; 200 nM). **Inset:** Western blot showing effectiveness of ERβ siRNA (200 nM for 48 hrs) tested in exponentially growing Daoy cells. Data represent average values from 3 experiments in triplicate (n = 9) with standard deviation. *indicate values significantly different from Cis (paired student t-test P≤0.05). **Panel B:** Evaluation of cell viability (ViaCount) in three medulloblastoma (Daoy, D283Med and D384Med) and two breast cancer (MCF7 and BT-20) cell lines. The cells were cultured in 10%FBS (FBS); 10%FBS+ICI182,780 (10 µM) (ICI); 10%FBS+Cisplatin (1 µg/ml) (Cis); and 10%FBS+ICI182,780 (10 µM) + Cisplatin (1 µg/ml) (Cis+ICI) for 48 hrs. Data represent average values from 2 experiments in triplicate (n = 6) with standard deviation. *indicate values significantly different from Cis (paired student t-test P≤0.05). **Panel C:** Evaluation of cell viability (ViaCount) in exponentially growing Daoy cells (10%FBS) treated with different doses of ICI182,780 ranging from 10 nM to 100 µM.

Previous studies indicate that the inhibition of ERβ may have anti-tumoral potential against different malignant neoplasms [12], [15], [16], [17] including Medulloblastomas [8]. To further analyze this possibility, we have selected human medulloblastoma cell lines, Daoy, D283Med and D384Med, which express high levels of ERβ in the absence of ERα [9], and asked if the effectiveness of cisplatin treatment could be enhanced by the ER antagonist, ICI182,780 [18], [19]. Surprisingly, our initial morphological evaluation, depicted in Fig. 1A, show only limited nuclear damage (typical for cisplatin treatment; arrowhead), which was accompanied by mitotic figures (asterix), when the cultures of Daoy cells were exposed to cisplatin (1 µg/ml) in the presence of 10 µM ICI182,780. Further analyses based on cell membrane permeability (ViaCount) and apoptotic DNA damage (TUNEL) confirmed ICI182,780-mediate protection of Daoy cells from the cisplatin induced cytotoxicity (Fig. 1B). Quantitatively, an average cell viability increased from 47.8+/−8.4% to 67.9+/−5.1% when the cell were exposed to cisplatin or to cisplatin+ICI182,780, respectively (45% increase in cell survival). In the same culture conditions, the percentage of apoptotic cells (TUNEL positive) decreased from 15.4+/−2.1% in the presence of cisplatin to 5.5+/−0.6% in the presence of cisplatin+ICI182,780 (Fig. 1B, lower panel). In addition, results in Fig. 2A demonstrate that ICI182,780 used at concentrations ranging from 10 nM to 10 µM protected Daoy cells from cisplatin-induced cell death. In a similar manner, siRNA against ERβ counteracted cisplatin-induced cytotoxicity (last bar in Fig. 2A), further indicating that ERβ is involved in ICI182,780-mediated cell protection from cisplatin. Another two human medulloblastoma cell lines, D384Med and D283Med, tested in the same condition showed 44.3% (significant) and 21.1% (not significant) increase in cell viability, respectively (Fig. 2B). A similar trend in cell survival was also observed in two breast cancer cell lines BT20 and MCF7, which are both known to express ERβ [20]. However, effects of ICI182,780 counteracting cisplatin-induced cytotoxicity was less pronounced, most likely because these two breast-cancer cell lines are significantly less sensitive to the cisplatin treatment (Fig. 2B). Interestingly, we did not observed any major effects of ICI182,780 on cell survival when tested, in the absence of cisplatin, in exponentially growing Daoy cells (10%FBS) at concentrations ranging from 10 nM to 100 µM (Fig. 2C).

### Inhibition of ER affects cisplatin-induced DNA damage checkpoints

**Figure 3:**
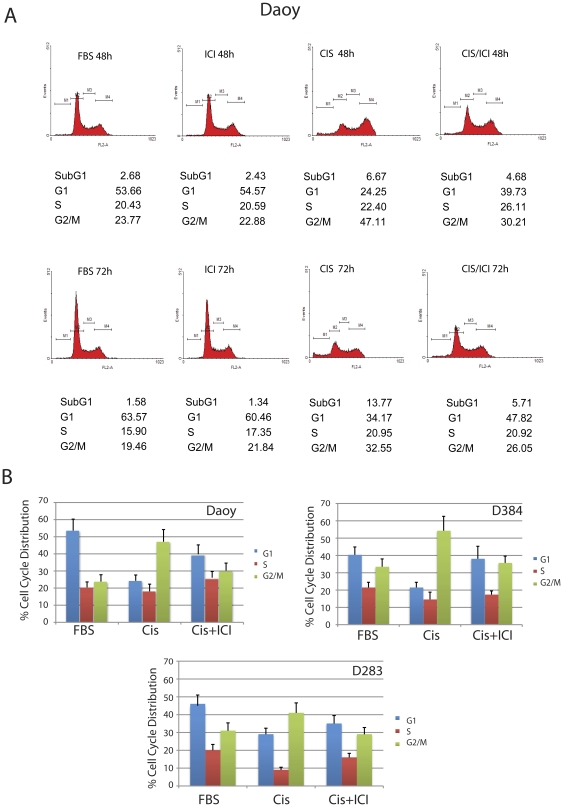
Effects of ICI182,780 on cell cycle distribution in cisplatin treated Daoy and D384Med cells. Exponentially growing cultures of Daoy, D384Med and D283 Med cells were treated with cisplatin (0.25 µg/ml) or with cisplatin + ICI182,780 (10 µM) for 24 (not included), 48 and 72 hours. Aliquots of 1×10^6^ cells/ml were fixed in 70% ethanol; the cells were centrifuged, labeled with propidium iodide/RNaseA solution and evaluated by Calibur flowcytometer and WinMDI 2.9 software. **Panel A:** Diagrams of cell cycle distribution (Daoy) from one representative experiment, which was repeated three times with a similar outcome. **Panel B:** Average data of cell cycle distribution (G1, S, G2/M) for Daoy, D384Med and D283Med cells with standard deviation (n = 3). Note the presence of a reproducible shift from G2/M to G1 cell cycle arrest between cisplatin-treated and cisplatin+ICI182,780-treated cells at 48 hrs time point.

The analysis of cell cycle distribution demonstrated a gradual shift from G2/M arrest, which usually happens in cisplatin-treated cells [21], to G1 arrest when the cells treated with cisplatin were cultured in the presence of ICI182,780 (ICI; Fig. 3A). This transition in cisplatin-induced cell cycle arrest is already visible at 24 hours (not shown), and became much more apparent at 48 hours time point in which G2/M fraction decreased from 47.1% (cisplatin only) to 30.2% (cisplatin+ICI) and G1 fraction increased from 24.2% (cisplatin only) to 39.7% (cisplatin+ICI). Importantly, the continuous cell exposure to cisplatin and ICI182,780 for 72 hours resulted in two-fold lower level of SubG1 fraction, which represents the population of necrotic and apoptotic cells (decrease from 13.8% in cisplatin to 5.7% in cisplatin+ICI; Fig. 3A, lower panel). We have repeated evaluation of cell cycle distribution in Daoy, D384Med and in D283Med cells several times and the average data are presented in Fig. 3B. Again, all cell lines examined show an apparent shift from G2/M to G1 cell cycle arrest when the cisplatin treatment is accompanied by ICI182,780-mediated inhibition of ERβ.

**Figure 4:**
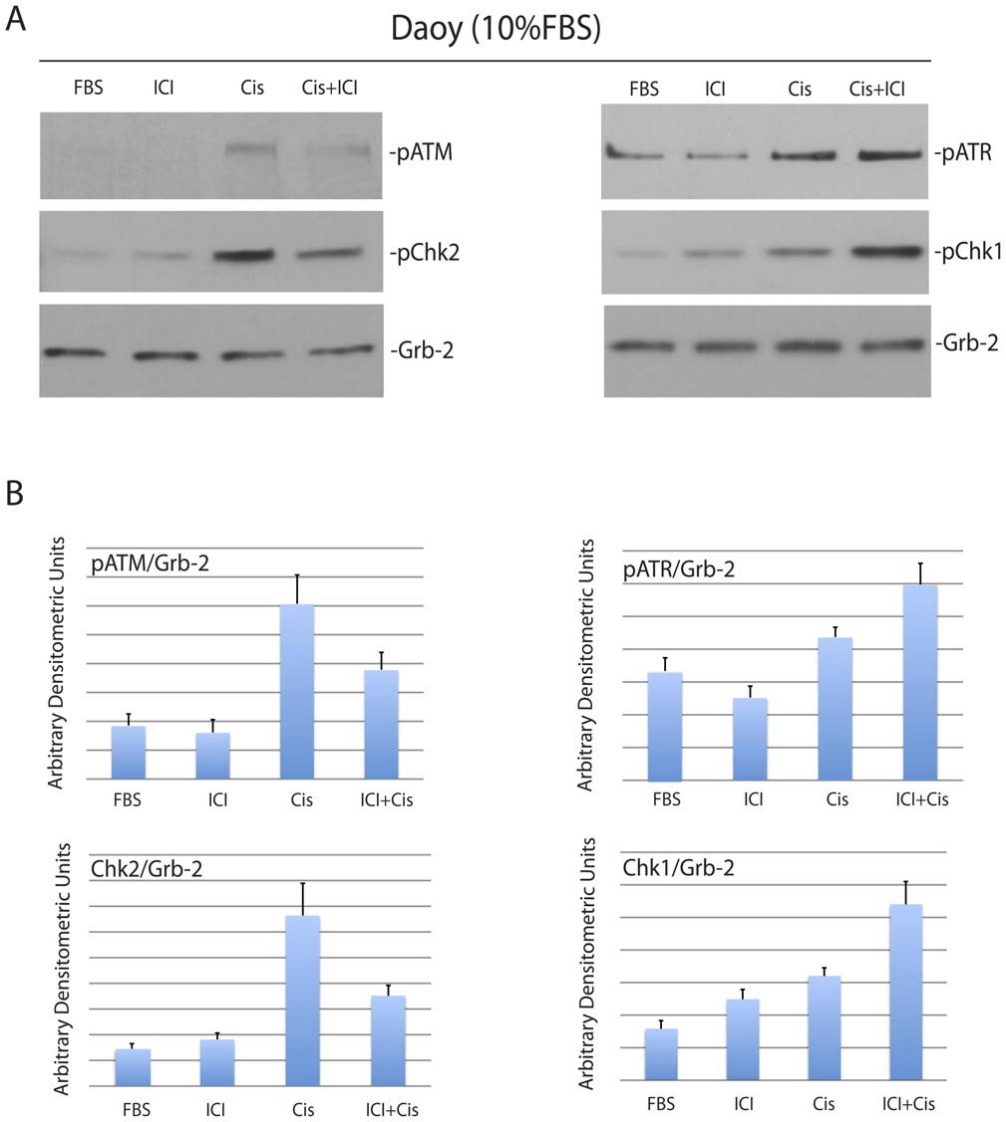
Inhibition of ERβ modulates cisplatin-induced phosphorylation of cell cycle checkpoint proteins. **Panel A:** Western blot analyses showing levels of the phosphorylated ATM, ATR, Chk1 and Chk2 in constitutively growing Daoy cells (10%FBS) treated with cisplatin (1 µg/ml) in the presence (Cis+ICI) or absence (Cis) of ICI182,780 (10 µM). The cells without treatment (FBS), or cells treated with ICI182,780 only (ICI) were used as controls. **Panel B:** Densitometry of Western blots depicted in Panel A evaluated by EZQuant-Gel 2.17 software. Levels of pATM, pATR, pChk1 and pChk2, were normalized with the corresponding levels of Grb-2. Data represent averages obtained from densitometric measurements of 3 blots with standard deviation and each band was normalized with corresponding loading control, Grb-2.

If indeed this transition in cell cycle distribution is based on DNA damage/cell cycle checkpoint system, we should observe also a shift in the phosphorylation pattern between ATM/Chk2 and ATR/Chk1 [22]. Of note, the cisplatin treatment is expected to trigger G2/M arrest followed by elevated apoptosis [23]. The results in Fig. 4 demonstrate very low levels of phosphorylation of ATM, ATR, Chk1 and Chk2 in the absence of DNA damage (FBS and ICI). Following the treatment with cisplatin (Cis), over 4-fold increase in ATM/Chk2 phosphorylation and 1.4-fold increase of ATR/Chk1 phosphorylation were observed after 6 hours. The phosphorylation pattern between ATM/Chk2 and ATR/Chk1 was reversed when the cisplatin treated cells were compared to the cells treated with cisplatin + ICI182,780. Quantitatively, ATM/Chk2 phosphorylations decreased by an average of 2-fold and ATR/Chk1 phosphorylations increased by an average of 1.5-fold (Fig. 4B). These results demonstrate that the transition from G2/M to G1 arrest observed in the presence of ICI182,780 was indeed accompanied by the transition from ATM/Chk2 to ATR/Chk1 activation.

### The cells preconditioned with ICI182,780 show more effective DNA repair and less DNA damage

**Figure 5:**
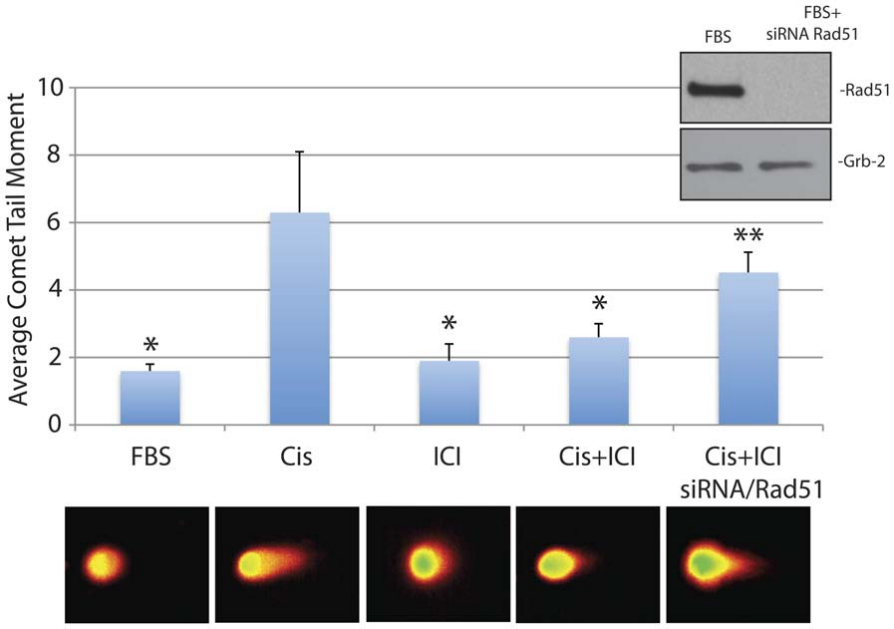
Inhibition of ERβ decreases cisplatin-induced DNA damage. Neutral comet assay (single cell electrophoresis) of exponentially growing Daoy cells (FBS) in which cisplatin treatment (1 µg/ml for 6 hours) was applied in the absence (Cis) or in the presence of 10 µM ICI182,780 (Cis+ICI). The histogram represents average Olive tail moment (with standard deviation) calculated from three experiments in duplicate (n = 6). In each experiment at least 100 cells were selected for the calculation (Automated Comet Assay; Loats Associates. Inc.). * indicates value statistically different from the sample labeled Cis. ** indicates value statistically different form Cis+ICI (paired student t-test; P≤0.05). **Inset:** Western blot showing effectiveness of Rad51 siRNA (100 nM for 48 hrs) tested in exponentially growing Daoy cells.

**Figure 6:**
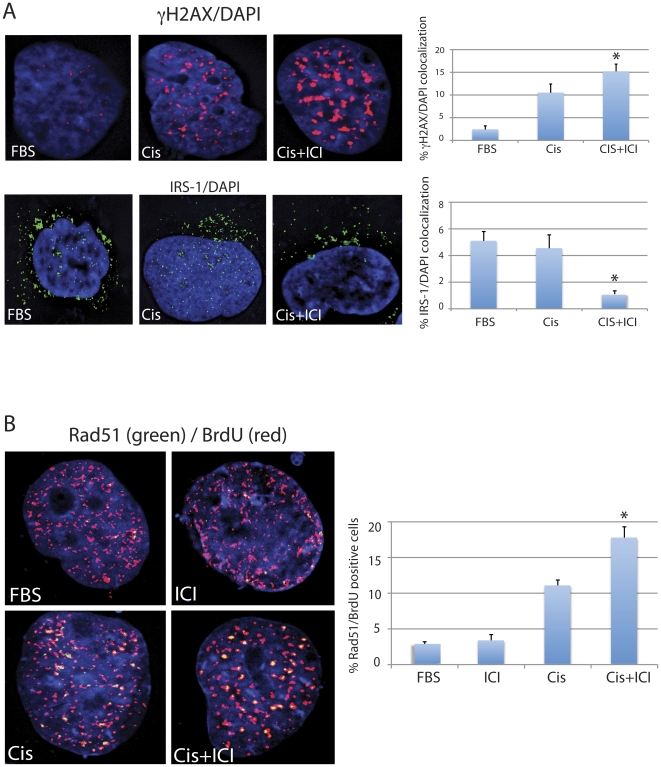
Inhibition of ERβ activates recruitment of Rad51 during S phase DNA repair. **Panel A:** Fluorescent images of Daoy cells immunolabeled with anti-histone γH2AX (upper panel) and anti-IRS-1 (lower panel) antibodies. The nuclei are visualized by DAPI staining (blue fluorescence). The histograms represent quantification of the co-localization between γH2AX and DAPI; IRS-1 and DAPI. The data represent average percentage of nuclear voxels (3-D pixels) of γH2AX (red fluorescence) and IRS-1 (green fluorescence) calculated from three independent experiments (n = 3) in which ten randomly selected cells have been evaluated by the Mask analysis included in SlideBook 5 deconvolution software. * indicates value statistically different from the sample labeled Cis (paired student t-test; P≤0.05). **Panel B:** Fluorescent images of the cells labeled with anti-Rad51 (green fluorescence) and with anti-BrdU (red fluorescence) antibodies. Exponentially growing cultures of Daoy cells (10%FBS) were exposed for one hour to bromodeoxyuridine (BrdU) during the 6 hours treatment with cisplatin (1 µg/ml) in the absence (Cis) or in the presence of 10 µM ICI182,780 (Cis+ICI). The histogram represents quantification of Rad51 positive cells in which Rad51 nuclear foci co-localize with BrdU-labeled DNA. Note, almost 40% increase in the number of cells utilizing Rad51 to repair cisplatin-induced DNA damage (during DNA replication) when the cisplatin treatment is accompanied by ICI182,780. * indicates value statistically different from the sample labeled Cis (paired student t-test; P≤0.05).

Since cellular responses to cisplatin and ICI182,780 are similar in all cell lines examined, we have selected Daoy cells to explore molecular basis of ICI182,780–induced resistance to cisplatin. In addition, effects of cisplatin and ICI182,780 were evaluated in cells replicating DNA (10%FBS), therefore the cisplatin treatment which primarily generates DNA-adducts and oxidative DNA damage [24], [25], [26] is also expected to cause DNA double strand breaks (DSBs) [27], [28], [29]. This happens when the replication forks are stalled on the cisplatin-induced primary DNA lesions [29], [30], [31]. We have used neutral comet assay to evaluate DSBs formation in Daoy cells treated with cisplatin [32]. The results in Fig. 5 show the average comet tail moment of 1.6+/−0.2 in control Daoy cells cultured in the presence of 10% FBS (FBS). The treatment with ICI182,780 (ICI) slightly increased this parameter to 1.9+/−0.5 (not significant). The average tail moment increased almost 4-fold (from 1.6+/−0.2 to 6.3+/−2) following cell exposure to 1 µg/ml of cisplatin (Cis). Importantly, a significant (*) 2.4-fold decrease in the tail moment (from 6.3+/−0.2 to 2.6+/−0.4) was observed when the Daoy cells were treated with cisplatin in the presence of ICI182,780 (Cis+ICI). This 2.4-fold decrease in the comet tail moment in the presence of ICI182,780 may suggest that either cisplatin generates less DNA damage, or that cisplatin-treated cells repair DSBs more effectively following the inhibition of ERβ. To address this question, we have utilized siRNA strategy against Rad51 – the major DNA repair protein involved in DSBs DNA repair during S-phase of the cell cycle [29], [33], [34]. The comparison between last two bars in Fig. 5 demonstrates that ICI182,780 is not able to rescue Rad51-deficient Daoy cells from cisplatin; note a significant increase (**) in comet tail moment from 2.6+/−0.4 to 4.5+/−0.6 (Fig. 5). Additionally, results in Fig. 6 show detectable changes in the phosphorylation pattern of histone H2AX (γH2AX - DNA damage response protein, which becomes phosphorylated within mega-basepare regions surrounding DNA strand breaks [28]). The number of γH2AX nuclear foci is relatively small in untreated exponentially growing Daoy cells (FBS), which increased dramatically in the presence of cisplatin (Fig. 6A; compare FBS and Cis). Despite of an apparent decrease in the DNA damage evaluated by the neutral comet assay (Fig. 5), the cells treated with cisplatin in the presence of ICI182780 (Cis+ICI) show an increase in γH2AX nuclear foci (evaluated by γH2AX/DAPI co-localization), which may imply more effective recruitment of DNA repair proteins, including Rad51 [35]. Of note, we have previously reported that ICI182,780–mediated inhibition of ERβ prevented translocation of IRS-1 to the nucleus and the binding between IRS-1 and Rad51 after DNA damage [9]. Indeed, results in Fig. 6A (lower panel) confirmed that only a small fraction of nuclear IRS-1 was detected in Daoy cells treated together with cisplatin and ICI182,780, which according to our previous observation is expected to increase the fraction of Rad51, which in the absence of nuclear IRS-1 can be recruited more effectively to the sites of DSBs, supporting HRR [9], [36]. The results in Fig. 6B show that cells in which cisplatin-induced DNA damage was accompanied by ICI182,780 treatment have significantly greater areas in which Rad51 co-localizes with the sites of DNA labeled by BrdU (*de novo* DNA replication). Quantitatively, the number of cells, in which 10 or more Rad51 nuclear foci co-localized with BrdU, increased almost 40% in the presence of ICI182,780 (Fig. 6B, histogram).

**Figure 7:**
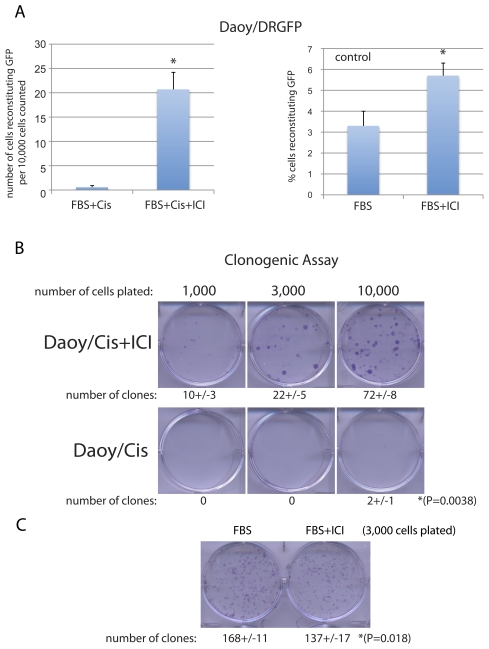
Inhibition of ERβ improves homologous replication directed DNA repair (HRR) and increases clonogenic growth of Daoy cells treated with cisplatin. **Panel A:** HRR was evaluated by the assay based on the reconstruction of the wild type green fluorescent protein (GFP) from two non-functional heteroallelic fragments of GFP cDNA delivered into cells by the pDRGFP expression vector [37]. HRR was evaluated in Daoy/DRGFP cells following transient transfection with the expression vector coding for I-*Sce-I* (rare cutting endonuclease), to inflict DNA double strand break in GFP cDNA, and with mito-red containing expression vector (control for the efficiency of transfection). The results were collected from three separate experiments in duplicate (n = 6) in which about 10,000 transfected cells *per* experiment were counted in at least ten randomly selected microscopic fields. * indicates value statistically different from the sample labeled Cis (paired student t-test; P≤0.05). The histogram labeled “DRGFP control” illustrates baseline HRR in exponentially growing Daoy cells in 10%FBS and in 10%FBS+10 µM ICI182,780. **Panel B:** Clonogenic assay. The monolayer cultures of Daoy cells were exposed to cisplatin (0.25 µg/ml) in the presence and in the absence of 10 µM ICI182,780 for 24 hours. Next, the medium containing cisplatin was replaced with the fresh medium and the cells were plated at the clonal-density (ranging from 1×10^3^ to 1×10^4^ cells per 35 mm dish) in the presence of 10%FBS. Clonogenic growth was evaluated after 14 days of a continuous cell growth as described in our previous work [50]. In control conditions (Panel C), the cisplatin treatment was omitted. The data represent average number of clones with standard deviation calculated from three independent experiments in duplicate (n = 6) *indicates values statistically different (paired student t-test; P≤0.05).

To evaluate if this significantly higher level of Rad51/BrdU co-localization correlates with increased HRR activity, we used previously generated in our lab Daoy/DRGFP cells [9], which stably express the HRR reporter cassette (DRGFP) [37]. Results in Fig. 7A demonstrate over 20-fold difference in HRR when the cisplatin treated Daoy/DRGFP cells were compared to Daoy/DRGFP cultured in the presence of cisplatin+ICI182,780. In particular, we have detected an average of 21+/−4 cells capable of repairing the DRGFP reporter cassette per 10,000 cells (n = 3); when the cisplatin treatment was accompanied by ICI182,780. In the absence of ICI182,780, we have detected only 1+/−1 cells capable of reconstituting the DRGFP per 10,000 cells (n = 3) (Fig. 7A; left panel). Note that in the absence of cisplatin (DRGFP control) the average level of HRR–mediated reconstitution of the functional GFP is about 3% in exponentially growing Daoy cells (10%FBS), which increased up to 5% in 10%FBS+ICI182,780 (Fig. 7A right panel, and [9]). Importantly, this ICI182,780-induced increase in HRR in cells treated with cisplatin correlated well with increased clonogenic growth of Daoy cells evaluated after the removal of cisplatin (Fig. 7B). In this experiment, we have used cisplatin at lower concentration (0.25 µg/ml) and analyzed its effects in the presence and absence of 10 µM ICI182,780. Following 24 hours, the cisplatin-containing culture medium was removed and the cells were re-pleated at 1,000; 3,000; and 10,000 cells/35 mm dish. The clonogenic growth was measured after 2 weeks of the continuous cell growth in the presence of 10%FBS. The results in Fig. 7B show that 24 hours of cell exposure to 0.25 µg/ml of cisplatin inhibited almost completely their future clonogenic growth. In contrast, Daoy cells treated with cisplatin in the presence of ICI182,780 formed an average of 10+/−3, 22+/−5 and 72+/−8 clones when plated at 1,000; 3,000; and 10,000 cells, respectively. Interestingly, in the absence of cisplatin, clonogenic growth of Daoy cells was significantly attenuated in cultures exposed to 10 µM ICI182,780 (Fig. 7C).

## Discussion

Results of this study demonstrate that human medulloblastoma cell lines develop resistance to cisplatin in the presence of a potential anticancer drug, estrogen receptor (ER) antagonist, ICI182,780. This unexpected finding involves a switch from cisplatin-induced G2/M arrest to G1 arrest accompanied by the activation ATR/Chk1 and inhibition of ATM/Chk2 - DNA-damage/cell cycle arrest pathway. In addition, cells exposed to cisplatin and ICI182,780 show elevated recruitment of Rad51 to the sites of damaged DNA and improved DNA repair by homologous recombination (HRR). This improved S phase DNA repair is considered to be responsible for a switch in cisplatin-induced cell cycle arrest from G2/M to G1 checkpoint, which correlates with better cell survival and partially preserved clonogenic growth. Our interpretation of the acquired resistance to cisplatin is based on the inhibition of ERβ-mediated translocation of IRS-1 to the nucleus [9]. In the absence of nuclear IRS-1 the recruitment of Rad51 to the sites of damaged DNA is not disturbed, therefore, Rad51 can support more effectively DNA repair by homologous recombination [36], [38]. This enhanced S phase DNA repair can explain also much lower fraction of cells arrested in G2/M, and transition in cell cycle distribution from G2/M to G1 arrest, when the cisplatin treatment is accompanied by ICI182,780 (Fig. 3). However, a different interpretation could be also possible. Recent work by Pedram et al. indicates that ERs agonist, 17-β-estradiol (E2), inhibited ATR/Chk1 in MCF7 breast cancer cells [39]. In addition, E2-treated MCF7 cells were characterized by delayed resolution of γH2AX phosphorylation, decreased Rad51 nuclear foci formation and less effective DNA repair [39]. Therefore, one could speculate that in contrast to ER activation, ER inhibition should improve the contribution of Rad51 to DNA repair. Indeed, this is what we have observed in medulloblastoma cells treated with both ICI182,780 and cisplatin (Fig. 6). Moreover, our data show that the inhibition of ERβ leads to enhanced ATR/Chk1 phosphorylation and the expected transition from G2/M to G1 cell cycle arrest [22], which was associated with better survival of medulloblastoma cells in the presence of cisplatin (Figs. 1 and 2). In MCF7 cells, E2-mediated stimulation of ERα and ERβ was accompanied by a decrease in ATR/Chk1 function towards G2/M arrest, which coincided with less effective DNA repair and increased chromosomal damage [39]. Again, the major difference here is that MCF7 cells express high levels of ERα and detectable levels of ERβ [9], [40], on the other hand, medulloblastoma cells are characterized by high levels of ERβ and practically undetectable ERα [9]. Therefore, DNA damage in MCF7 cells in which ERα and ERβ were activated lead to the inhibition of G2/M checkpoint, which resulted in less effective DNA repair. In our case however, DNA damage in medulloblastoma cells in which ERβ was inhibited, resulted in transition from G2/M to G1 checkpoint, better DNA repair, and improved cell survival, which attenuated cytotoxic action of cisplatin.

In view of these results and in respect to anticancer treatment, ICI182,780, has been already proposed for hormone sensitive breast cancer especially when the tumor cells develop resistance to tamoxifen, or to avoid tamoxifen-mediated partial agonistic side effects in estrogen-sensitive tissues such as endometrium and uterus [41]. It has been shown also that in difference to tamoxifen, ICI182,780 binds and inactivates ERα and ERβ without any agonistic effects on these nuclear receptors [18], [42]. Although the role of ERα in several tumors, including breast, ovarian, prostate and colon cancer has been intensively studied, a potential function of ERβ in malignant transformation is still unclear. ERβ has been detected in breast, ovarian, prostate and colon cancer, and in CNS tumors including glioblastoma and medulloblastoma [15], [16], [43], [44]. In some of these cancers, ERβ levels decline in close correlation with the development of less differentiated phenotype [43], [45], [46], which correlates well with our previous finding of nuclear ERβ in well-differentiated desmoplastic and neuroblastic medulloblastoma [9]. Additionally, there is an increasing tendency of using ICI182,780 in combination with other hormonal, cytotoxic, or genotoxic therapies. For instance, combine treatment with ICI182,780 and cisplatin demonstrated a strong synergistic action against ovarian [18] and cervical [19] cancer cells in vitro. Interestingly, several reports indicate that anticancer activities of ICI182,780 have been observed also in cancer cells which are ERα negative [17], [18], [47], [48]. This may imply the involvement of cellular reactions to the inhibition of ERβ, which are not fully understood and are suspected to be very different from those, which are related to the inhibition of ERα. In this respect, inhibition ERβ could have an important impact on medulloblastoma in which ERβ protein levels are high, and levels of ERα are either very low or practically undetectable [8], [9]. For instance, recently published results by Belcher et al. [8] demonstrate that activation of ERβ in human medulloblastoma cell line, D283Med, resulted in both increased cell growth and cell migration, and that ICI182,780 attenuated medulloblastoma tumor growth in the mouse model based on subcutaneous injection of D283Med cells. Our present work indicates, however, that 10 µM ICI182,780 had only a modest inhibitory action on D384Med medulloblastoma cells, and partially attenuated clonogenic growth of Daoy cells (Fig. 7B). This particular concentration of ICI182,780 was selected because it inhibited ERβ transcriptional activity in three previously tested medulloblastoma cell lines [9], and was used in several studies involving prostate cancer cell lines [11]. We are not certain why in our experimental setting Daoy and D384Med are much less sensitive to ICI182,780 treatment since they express ERβ at the levels comparable to D283Med, and are all practically negative for ERα [8], [9]. The only obvious difference is the concentration of ICI182,780, which in our studies is 100-times higher than in the experiments presented by Belcher et al. [8], [9]. Nerveless, Daoy, D384Med and to the lesser extent D283Med acquire resistance to cisplatin when this genotoxic agent is used together with 10 µM ICI182,780 (Fig. 2B). This unexpected side effect observed in cell lines should be carefully examined in view of the increasing number of preclinical studies in which combine treatment of cisplatin and ICI182,780 are proposed.

For instance, different cellular responses have been observed in ovarian [18] and cervical cancer cells [19] in which ICI182,780 improved genotoxic action of cisplatin. These apparent discrepancy in cellular responses to cisplatin + ICI182,780 treatment may suggest that different cellular context have to be considered during the selection of ICI182,780 as a supplemental drug for a particular anticancer therapy. Since ICI182,780 inhibits both ERα and ERβ, and these two nuclear receptors mediate different and often opposite cellular responses, better understanding of ERβ and its role in normal and pathologic growth of neural progenitors is absolutely required before pharmacological manipulations targeting this nuclear receptor could be used as a clinical regimen against medulloblastoma. Our present findings suggest for instance that the combined cisplatin and ICI182,780 treatment may predispose medulloblastoma cells to recurrences after the genotoxic treatment is completed.

## Materials and Methods

### Cell culture

We have used three human medulloblastoma cell lines, Daoy, D384Med and D283Med. Daoy derive from a tumor in the posterior fossa of a 4 years-old boy (ATCC# HTB186), D283Med (ATCC#HTB-185), and D384Med [49] are metastatic medulloblastomas isolated from peritoneal ascites of children diagnosed with medulloblastoma. Daoy were maintained as monolayer cultures in Dulbecco's Modified Eagle Medium (DMEM) (GIBCO-BRL, Grand Island, NY) containing 10% fetal bovine serum (FBS), at 37°C in a 7% CO_2_ atmosphere. D283Med and D384Med were cultured in suspension in DMEM supplemented with non-essential amino acids (GIBCO-BRL, Grand Island, NY), 2 mM L-glutamine, 1 mM sodium pyruvate, and 10% FBS. We have used MCF7 (ATCC# HTB-22) and BT-20 (ATCC# HTB-19) human breast cancer cell lines as a reference point in the experiment depicted in Fig. 2B. Exponentially growing cells were treated with cisplatain at 0.25 and 1.0 µg/ml in the presence or absence of ERβ antagonist, ICI182,780 (10 nM-100 µM; Tocris Bioscience, Ellisville, Mo) [9]. In some experiments, expression of Rad51 and ERβ was inhibited by utilizing ON-TARGETplus SMARTpool siRNA against human Rad51 - target sequences: CCAACGAUGUAAGAAUU; GCAGUGAUGUCCUGGAUAA; CUAAUCAGGUGGUAGCUCA; UAUCAUCGCCCAUGCAUCA (100 nM, Thermo Scientific); and human ERβ - target sequences: GGAAAUGCGUAGAAGGAAU; UUCAAUUUCGAGAGUUA; GCACGGCUCCAUAUACAUA; GAACCCACAGUCUCAGUGA (200 nM; Thermo Scientific) delivered to the cells by Oligofectamine transfection reagent (Invitrogen).

### Cell cycle distribution, DNA replication and Cell viability

We have used GUAVA easyCyte 8HT and Calibur flow cytometers to detect and quantify these three cellular parameters. Briefly, the aliquots of 1×10^6^ cells/ml were fixed in 70% ethanol at −20°C, overnight. The cells were centrifuged at 1,600 rpm and the resulting pellets suspended in 1 ml of freshly prepared Propidium Iodide/RNaseA solution. Cell cycle distribution was evaluated using specialized software CellCycle included in GuavaSoft 1.1. In some experiments DNA replication was evaluated by BrdU pulse labeling (1 hour) using the DNA replication Assay (Millipore). Finally, cell death and cell survival were evaluated by two independent assay, TUNEL assay (Roche), which detects DNA damage associated with apoptosis, and cell membrane integrity by using ViaCount reagents, according to the manufacturer recommendations. Guava/Express plus and Guava/ViaCount software were used for data analyses.

### Neutral Comet Assay (single cell electrophoresis)

Was utilized to detect DNA strand breaks in exponentially growing Daoy cells exposed to cisplatin in the presence and absence of ICI182,780. The cells treated with H2O2 (oxidative DNA damage) or neocarzinostatin (NCS; Sigma, Saint Louis, MO) were used as positive controls for the detection of secondary and primary DNA strand breaks (DSBs), respectively. The cells were subjected to single cell electrophoresis in neutral conditions [32] by utilizing SYBR green based kit from Trevigen and Automated Comet Assay System from Loats Associates Inc. The tail moment was calculated from 100 cells collected per single measurement by utilizing specialized comet software included in the Automated Comet Assay System (Loats Associates Inc., Westminster, MD).

### Western Blot

To isolate protein extracts, monolayer cultures were treated with 400 µl of lysis buffer A [50 mM HEPES; pH 7.5; 150 mM NaCl; 1.5 mM MgCl_2_; 1 mM EGTA; 10%glycerol; 1% TritonX-100; 1 mM phenylmethylsulfonyl fluoride (PMSF); 0.2 mM Na-orthovanadate and proteinase inhibitor cocktail] on ice for 5 minutes. Total proteins (50 µg) were separated on a 4–15% gradient SDS-PAGE (BioRad). The resulting blots were probed with following primary antibodies: anti-pSer1981 ATM mouse monoclonal antibody (Cell Signaling Technology Inc., Danvers, MA); anti-pSer428 ATR rabbit polyclonal antibody (Cell Signaling Inc.); anti-pSer317 Chk1 rabbit polyclonal (Cell Signaling Inc.), anti-pThr68 Chk2 rabbit polyclonal (Cell Signaling Inc.). Anti-Grb-2 antibody (Transduction Laboratories, Lexington, KY), was used to monitor equal loading conditions [33].

#### Immunocytofluorescence

All cells were cultured on glass culture slides (BD Falcon, Franklin Lakes, NJ). Cisplatin treatment, 0.25–1 µg/ml, was applied to exponentially growing cells for a period of 6 hours. For immunostaining the cells were fixed and permeablized with the buffer containing 0.02% Triton X-100 and 4% formaldehyde in PBS. Fixed cells were washed 3× in PBS and blocked in 5% BSA for 1 hour at 37°C. RAD51 was detected by rabbit anti-RAD51 polyclonal antibody (Santa Cruz Inc., Santa Cruz, CA) followed by AlexaFluor-conjugated donkey anti-rabbit secondary antibody (Invitrogen, Carlsbad, CA). Phospho-histone H2AX (S139) was detected by a rabbit polyclonal antibody (UBI, Lake Placid, NY), and rhodamine-conjugated goat anti-rabbit secondary antibody (Molecular Probes). IRS-1 was detected by anti-IRS-1 mouse monoclonal antibody (Santa Cruz Biotechnology Inc., Santa Cruz, CA) followed by FITC-conjugated goat anti-mouse secondary antibody (Molecular Probes, Inc. Eugene, OR). DNA replication was monitored by labeling the exponentially growing cells with Bromodeoxyuridine (BrdU) followed by immunofluorescence with anti-BrdU antibody, according to manufacturer recommendations (DNA Replication Assay; Millipore). The images were visualized with the Nikon Eclipse E400 upright fluorescence microscope equipped with EXI aqua camera (Qimaging), motorized Z-axis, and SlideBook5 acquisition/deconvolution software (Intelligent Imaging Innovations, Inc., Denver, CO). A series of three-dimensional images of each individual picture were deconvoluted to one two-dimensional picture and resolved by adjusting the signal cut-off to near maximal intensity to increase resolution. Final pictures were prepared with Adobe Photoshop to demonstrate subcellular localization and co-localization between detected proteins. Quantification of colocalization between: Rad51 and DAPI; IRS1 and DAPI; Rad51 and BrdU were performed by utilizing Mask analysis included in SlideBook5 software according to manufacturer recommendation (Intelligent Imaging Innovations, Inc).

#### Clonogenic Growth

Exponentially growing cultures of Daoy cells (10%FBS) were either untreated (control) or treated with 0.25 µg/ml of cisplatin for 24 hours. The ICI182,780 pretreatment started 16 hours before cisplatin was applied and continued for an additional 24 hours in the presence of cisplatin. Next the cells were washed with fresh serum-free medium, trypsinized and transferred to 35 mm culture dishes at clonal-densities ranging from 1×10^3^ to 1×10^4^ cells. Clonogenic growth was evaluated 2 weeks after continuous cell growth in the medium containing 10%FBS and the resulting clones were fixed and stained with 0.25% Cristal Violet in methanol as described in our previous work [50].
